# Epidemiological Aspects, Prenatal Screening and Diagnosis of Congenital Heart Defects in Beijing

**DOI:** 10.3389/fcvm.2021.777899

**Published:** 2021-12-20

**Authors:** Yanchun Zhang, Wen Zhang, Hongyan Xu, Kaibo Liu

**Affiliations:** ^1^Perinatal Health Department, Beijing Obstetrics and Gynecology Hospital, Capital Medical University, Beijing, China; ^2^Perinatal Health Department, Beijing Maternal and Child Health Care Hospital, Beijing, China

**Keywords:** congenital heart disease, prenatal screening, prenatal diagnosis, detection rate, epidemiology

## Abstract

**Background:** In China, congenital heart disease (CHD) is the most common birth defect type, with approximately 13,000 new cases annually. This study aimed to investigate high-risk factors, prenatal screening and prenatal diagnosis as a basis for clinical decisions.

**Methods:** All CHD cases identified from 2018 to 2020 were obtained from the Beijing city birth defect surveillance system and prenatal diagnosis institutions. The prenatal CHD diagnosis was confirmed by fetal echocardiography and amniotic fluid or cord blood genetic examination. Chi-square, odds ratio (OR), 95% confidence interval (CI), and univariate and multivariate logistic analyses were used to explore the high-risk factors, prenatal screening and prenatal diagnosis of CHD. Results: In total, 6,786/594,860 fetuses with CHD were diagnosed by prenatal echocardiography. The average incidence of CHD was 11.4 per 1,000 births, with an increase of 30.7 per 1,000 births from 2018 to 2020 (*P* < 0.05); the average incidence of complex CHD (CCHD) was 2.02 per 1,000 births, with no significant change from 2018 to 2020 (*P* > 0.05). Women age ≥35 years (OR 1.06, 95% CI 0.77–1.46) was at higher risk of having babies with CHD than women aged 21–34 years. Overall, CHD incidence increased with maternal age (OR1.03, 95% CI 1.02–1.03). Additionally, women who had a non-local household registration (OR 1.16, 95% CI 1.10–1.22) or had diabetes mellitus (DM) (OR 1.16, 95% CI 0.96–1.25) were at higher risk of CHD. As an independent factor, CCHD was related to maternal age, DM, fetal gender, and maternal education level (all *P* < 0.05). The prenatal ultrasound screening detection rate of CCHD was 97.59%, which was far higher than that of total CHD (51.67%) (*P* < 0.001). The prenatal ultrasound diagnosis rate of CCHD was higher than that of simple CHD (*P* < 0.001), but the coincidence rate in the ultrasound diagnosis of CCHD was lower than that of simple CHD (*P* < 0.001). Prenatal genetic testing revealed chromosomal abnormalities in 25.62% (279/1089) of CHD cases with indications for a prenatal diagnosis.

**Conclusions:** Maternal age, household registration and DM were related to CHD occurrence. Prenatal ultrasound screening is a highly effective method for CCHD diagnosis, and CHD fetuses should be closely evaluated to exclude chromosomal abnormalities.

## Introduction

Congenital heart disease (CHD) refers to congenital malformations caused by single or multiple structural and functional developmental abnormalities in the heart or great vessels during the fetal period. It has been reported that the global average prevalence of CHD at birth was 8.2 per 1,000 births from 1970 to 2017 (1). Moreover, in China, CHD accounted for 26.7% of all birth defects in 2011 according to the “China Birth Defect Prevention Report (2012)” (2). CHD is the main cause of death among perinatal infants and infants under 5 years of age (3). In 2015, an estimated 303,300 infants died worldwide due to CHD (4). Epidemiological studies have shown that the early diagnosis of CHD and early intervention can significantly improve the prognosis of neonates with CHD (5). After the updated fertility policy was implemented, some changes in the characteristics of women having babies and associations with CHD risk began to emerge (6, 7). Therefore, it is necessary to characterize the detailed epidemiology of CHD, especially in Beijing, the capital of China. The purpose of this study was to comprehensively investigate the incidence, high-risk factors, prenatal ultrasound screening and diagnosis and genetic detection of CHD from 2018 to 2020 in Beijing city. Our research results may provide data supporting countries in formulating policies to prevent CHD.

## Materials and Methods

### Study Population

In Beijing, the birth defect surveillance system covers all regions and includes all midwifery hospitals. The registry system captures congenital anomalies in all births. A birth defect registration card was used by medical staff for data collection in the surveillance hospitals, and the information of women and their babies, birth defect diagnosis, and pregnancy test results were obtained from clinical records, all of which were available online. We performed a cross-sectional study of CHD reported in this surveillance system from 2018 to 2020.

### CHD Diagnosis

In this study, CHD was diagnosed using the International Classification of Diseases version 10 (ICD-10). Simple CHD refers to heart disease with a single defect that generally does not cause hemodynamic changes. We included atrial septal defects (ASDs) ≥3 mm and patent ductus arteriosus (PDA) ≥3 mm. We defined 12 types of CHD as CCHD according to the US CDC standards (8, 9), namely, persistent truncus arteriosus (PTA), double-outlet right ventricle (DORV), d transposition of the great vessels (DTGA), single ventricle (SV), tetralogy of Fallot (TOF), pulmonary valve atresia, Ebstein malformation, Tricuspid atresia, hypoplastic left heart syndrome (HLHS), coarctation of the aorta (COA), interrupted aortic arch (IAA), and total anomalous pulmonary venous return (TAPVR). The final diagnosis was based on echocardiography and confirmed by pediatricians. According to the above criteria, we artificially divided the simple CHD and CCHD cases into two groups according to whether they were combined with extracardiac malformations, followed by 1\2\3\4.

### Prenatal Ultrasound Screening and Diagnosis

**Figure 1:**
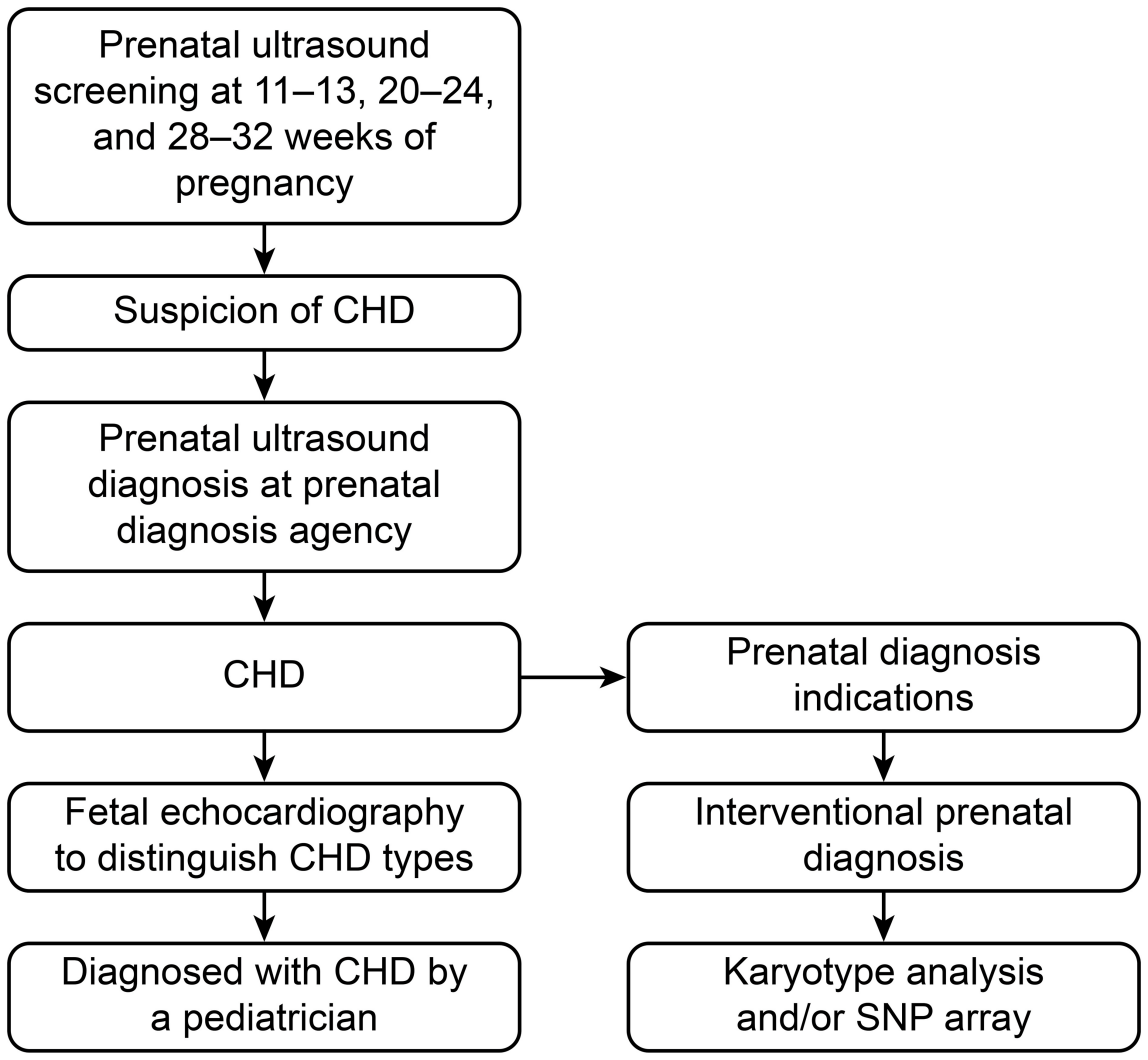
The process of prenatal ultrasound screening and diagnosis of CHD.

In Beijing, pregnant women are routinely screened by ultrasound at 11–13, 20–24 and 28–32 gestational weeks during pregnancy. If the ultrasound screening is abnormal, an ultrasound diagnosis is recommended. If there is an abnormal ultrasound finding, such as fetal nuchal translucency thickness over 3.0 mm or above the 95th percentile or structural abnormalities, an interventional prenatal diagnosis is recommended. The interventional prenatal diagnosis was based on villous tissue, amniotic fluid or cord blood used to perform a karyotype analysis and/or single nucleotide polymorphism (SNP)-array detection. The process of the prenatal screening and prenatal diagnosis of CHD is shown in Figure 1.

### Statistical Analysis

The data analyses were performed using SPSS statistical package version 18.0. The incidence and subtype of CHD are shown as the number of cases per 1,000 births. The definition of coincidence between prenatal ultrasound screening and diagnosis is that the main ultrasound results of the fetus are consistent between the ultrasound specialists with screening qualification and diagnosis qualification. The coincidence rate of prenatal ultrasound screening and diagnosis = the number of screening and diagnosis cases with consistent results/the total number of cases diagnosed by prenatal ultrasound. The quantitative data are shown as the mean ± standard deviation. The qualitative data are presented as numbers (*n*) and percentages (%). A chi-squared test was used to assess the between-group differences and determine the level of the association of the characteristics of pregnant women with CHD or CCHD. Univariate logistic regression models were fitted to identify the factors that were independently associated with CHD or CCHD. Only factors associated with CHD or CCHD found to be statistically significant were considered in the model building exercise using a multiple logistic regression model. The odds ratio (OR) and 95% confidence interval (CI) were calculated to examine the risk factors for CHD or CCHD. *P* < 0.05 was considered statistically significant.

## Results

### Basic Characteristics of Mothers With CHD Fetuses

In total, 594,860 babies were monitored for birth defects from January 2018 to December 2020. Among these, 6,786 fetuses (11.40‰) were diagnosed with CHD by systemic ultrasound and fetal echocardiography. In maternal age ≥35 years, the incidence of CHD was 17.01‰. Among the women with different household registrations, the incidence of CHD was higher in foreign households (13.13‰). Pregnant women with a bachelor's degree and above (12.10‰) and those with DM (12.46‰) had a higher incidence of CHD, and the birth of females (12.88‰) or multiple births (13.05‰) was higher than the birth of males or single births (all *P* < 0.05). The incidence of CHD among Han nationalities was slightly lower than that among other ethnic minorities, but there was no significant difference (Table 1).

**Table 1:** Basic characteristics of pregnant women with CHD fetuses.

**Variable**	**Births (*n*)**	**CHD (constituent ratio%)**	**Incidence (‰)**	***P*** **-value**
**Maternal age (years)**				
≤ 20	4,506	41 (0.60)	9.10	
21–34	459,202	4,682 (69.00)	10.20	<0.001
≥35	121,312	2,063 (30.40)	17.01	
**Household registration**				
Local	317,545	3,649 (53.77)	11.49	<0.001
Nonlocal	182,368	2,394 (35.28)	13.13	
**Nationality**				
Han	466,468	5,625 (82.89)	12.06	0.55
Non-Han	33,418	415 (6.12)	12.42	
**Education**				
< Bachelor's degree	113,650	1,082 (15.94)	9.52	
≥ Bachelor's degree	471,370	5,704 (84.06)	12.10	<0.001
**Birth gender**				
Male	308,410	3,098 (45.65)	10.05	<0.001
Female	286,437	3,688 (54.35)	12.88	
**Fetus number**				
Multiple birth	14,641	191 (2.81)	13.05	
Singleton birth	580,219	6,595 (97.19)	11.37	<0.001
**Diabetes mellitus**				
Yes	64,438	803 (11.83)	12.46	
No	530,422	5,983 (88.17)	11.28	<0.001

### Time Trends and Subgroups of CHD

**Figure 2:**
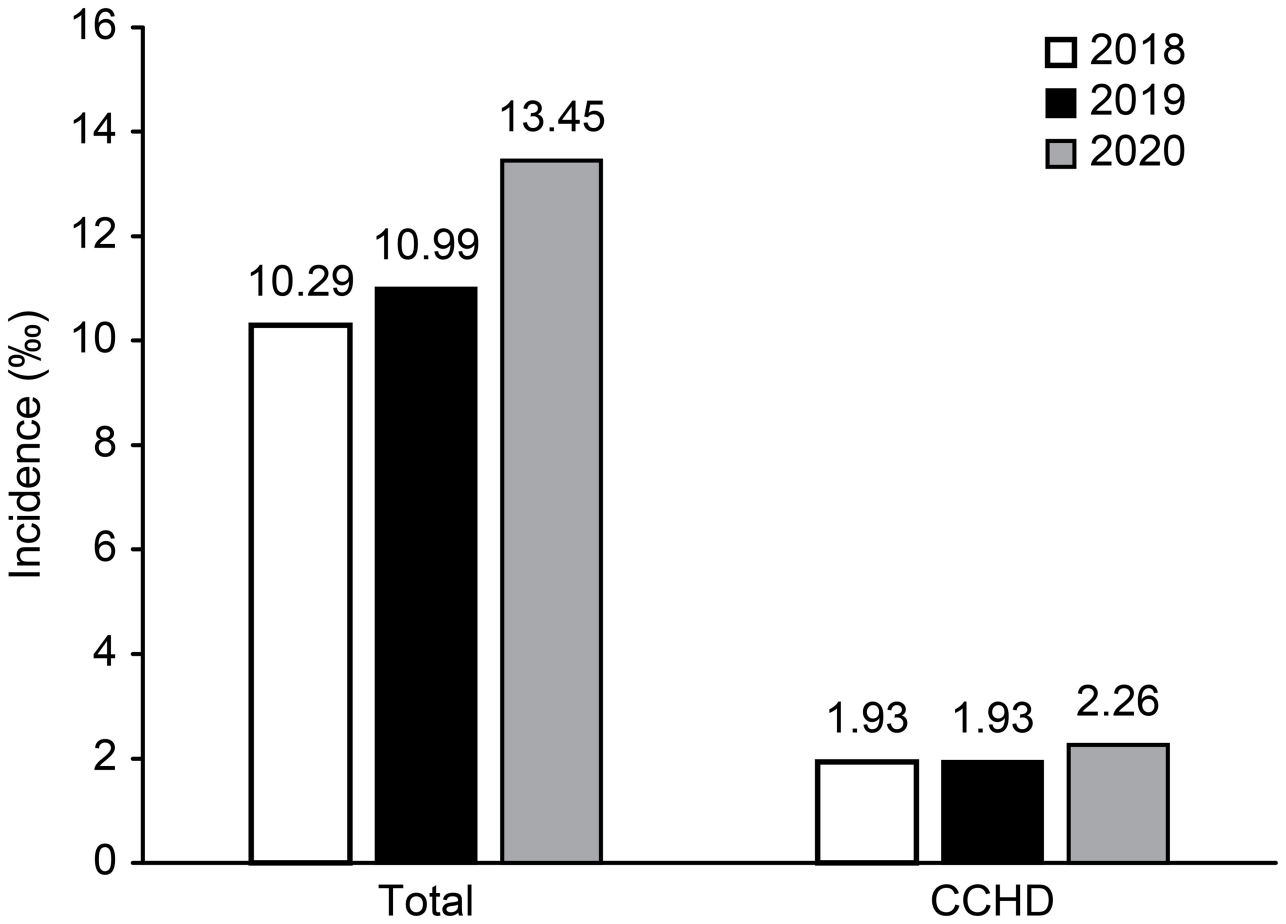
The incidence of CHD in Beijing from 2018 to 2020.

The overall incidence of CHD increased from 10.29‰ (2206/214333) in 2018 to 13.45‰ (2,174/161,605) in 2020, revealing an increasing trend of 30.7% [overall: χ^2^ = 50.13, *P* < 0.01; 2018 vs. 2019: χ^2^ = 4.94, *P* < 0.05); 2019 vs. 2020: χ^2^ = 23.24, *P* < 0.01; 2018 vs. 2020 (χ^2^ = 47.75, *P* < 0.01)]. However, 1,201 cases (2.02‰) were diagnosed with CCHD, and there was no significant change in the CCHD incidence over the previous three years (χ^2^ = 5.00, *P* = 0.08) (Figure 2).

Among 6,786 CHD cases, 4,763, 822, 934, and 267 cases were simple CHD (group 1), simple CHD plus extracardiac abnormality (group 2), CCHD (group 3), and CCHD plus extracardiac abnormality (group 4), respectively. The percentage of extracardiac abnormalities among the simple CHD cases was 14.72% (822/5,585), and that among the CCHD cases was 22.23% (267/1,201) (χ^2^ = 41.40, *P* < 0.001) (Table 2).

**Table 2:** Numbers (*N*) and proportions of CHD in the subgroups.

**Year**	**Total CHD (*N*)**	**Group 1**		**Group 2**		**Group 3**		**Group 4**	
		***N***	**Proportion**	***N***	**Proportion**	***N***	**Proportion**	***N***	**Proportion**
2018	2,206	1,510	68.45	283	12.83	326	14.78	87	3.94
2019	2,406	1,681	69.87	303	12.59	331	13.76	91	3.78
2020	2,174	1,572	72.31	236	10.86	277	12.74	89	4.09
Total	6,786	4,763	70.19	822	12.11	934	13.76	267	3.93

*Group 1 had simple CHD; Group 2 had simple CHD plus extracardiac abnormality; Group 3 had CCHD; Group 4 had CCHD plus extracardiac abnormality*.

Isolated ventricular septal defect (VSD), isolated ASD and isolated PDA accounted for 31.15% (2,114/6,786) of all CHD cases. The proportion of isolated ASD in CHD increased from 9.56% in 2018 to 15.64% in 2020, but the proportions of the other two subtypes decreased (PDA, 4.58 vs. 3.45%; VSD, 15.82 vs. 13.98%) (Table 3). In total, 292 CHD cases had multisystem malformations, accounting for 26.81% of CHD plus extracardiac abnormalities.

**Table 3:** Numbers (*N*) and proportions of isolated VSDs, ASDs and PDAs.

**Year**	**Total CHD (*N*)**	**Isolated VSD**		**Isolated ASD**		**Isolated PDA**	
		***N***	**Proportion**	***N***	**Proportion**	***N***	**Proportion**
2018	2,206	349	15.82	211	9.56	101	4.58
2019	2,406	380	15.79	259	10.76	95	3.95
2020	2,174	304	13.98	340	15.64	75	3.45
Total	6,786	1,033	15.22	810	11.94	271	3.99

### Risk Factor Analysis of CHD

The mean age of the pregnant women was 30.99 ± 4.71 years (range 13–68 years), and the mean age of the CHD mothers was 31.46 ± 4.36 years (range 16–54 years). Women aged ≥35 years (OR 1.06, 95% CI 0.77–1.46) was at a higher risk of having a baby with CHD than women aged 21–34 years (*P* < 0.01). Age, education level, birth gender and number, DM and household registration were independently associated with CHD. Using a multiple logistic regression model to analyze the above factors, age (OR 1.03, 95% CI 1.02–1.03), DM (OR 1.16, 95% CI 0.96–1.25) and household registration (OR 1.16, 95% CI 1.10–1.22) were related to CHD (Table 4). We divided CHD into simple CHD and CCHD. We used the same methods to fit a model for the risk factors for CCHD. Age (OR 1.03, 95% CI 0.96–1.11), education level (OR 1.25, 95% CI 1.05–1.49), DM (OR 1.05, 95% CI 0.96–1.11) and birth gender (OR 0.61, 95% CI 0.54–0.70) were also independently associated with CCHD. A multiple logistic regression model was used to analyze maternal age ≥35 years (OR 3.70, 95% CI 1.12–12.50) and male birth gender (OR 0.67, 95% CI 0.59–0.77) were related with CCHD (Table 5).

**Table 4:** Multiple logic regression model of the factors associated with CHD.

**Factor**	**Level**	**Unadjusted OR (95% CI)**	**Adjusted OR (95% CI)**	***P*** **-value**
**Maternal age (years)**		1.02 (1.01–1.02)	1.03 (1.02–1.03)	<0.001
	≤ 20	0.60 (0.57–0.63)	0.79 (0.74–0.84)	<0.001
	21–34	Ref	Ref	
	≥35	1.88 (1.38–2.57)	1.06 (0.77–1.46)	
**Household registration**				
	Local	Ref	Ref	<0.001
	Nonlocal	1.15 (1.09–1.20)	1.16 (1.10–1.22)	
**Education**				
	< Bachelor's degree	0.79 (0.74–0.84)	0.96 (0.90–1.03)	0.21
	≥Bachelor's degree	Ref	Ref	
**Birth gender**				
	Male	0.79 (0.74–0.84)	0.98 (0.93–1.03)	0.35
	Female	Ref	Ref	
**Fetus number**				
	Multiple birth	Ref	Ref	0.29
	Singleton birth	0.87 (0.75–1.00)	0.93 (0.80–1.07)	
**Diabetes mellitus**				
	Yes	1.11 (1.03–1.19)	1.16 (0.96–1.25)	<0.001
	No	Ref	Ref	

**Table 5:** Multiple logic regression model of the factors associated with CCHD between CHD and CCHD.

**Variable**	**CHD**	**CCHD**	***P*** **-value**	**Unadjusted OR (95% CI)**	**Adjusted OR (95% CI)**	***P*** **-value**
**Maternal age (years)**						
≤ 20	41	3		0.54 (0.47–0.61)	0.58 (0.51–0.66)	<0.001
21–34	4,682	693	<0.001	Ref	Ref	
≥35	2,063	505		4.11 (1.26–13.36)	3.70 (1.12–12.50)	0.03
**Household registration**						
Local	3,649	532	0.55			
Nonlocal	2,394	363				
**Nationality**						
Han	5,625	833	1.00			
Non-Han	415	61				
**Education**						
< Bachelor's degree	1,082	163		Ref	Ref	
≥ Bachelor's degree	5,704	1,038	0.01	1.25 (1.05–1.49)	1.14 (0.94–1.35)	0.18
**Birth gender**						
Male	3,098	431	<0.001	0.61 (0.54–0.70)	0.67 (0.59–0.77)	
Female	3,688	770		Ref	Ref	<0.001
**Fetus number**						
Multiple birth	191	24				
Singleton birth	6,595	1,177	0.07			
**Diabetes mellitus**						
Yes	803	148		1.05 (0.96–1.11)	1.16 (0.95–1.43)	
No	5,983	1053	0.02	Ref	Ref	0.15

### Genetic Examination of CHD

In total, 1,089 cases underwent interventional prenatal genetic examination; among these cases, chromosomal abnormalities were detected in 279 cases (25.62%). Among these 279 cases, 70 cases had trisomy 21, 62 cases had trisomy 18, 9 cases had trisomy 13, 26 cases had abnormalities in the structure or number of the sex chromosomes, and the other 112 cases had abnormalities in the number or structure of another chromosome. The abnormal karyotype detection rate in CCHD (7.16%) was significantly higher than that in simple CHD (3.46%) (χ^2^ = 33.5, *P* < 0.01), and the incidence of genetic abnormalities in the CHD patients with extracardiac abnormalities (11.96%) was significantly higher than that in the CHD patients without extracardiac abnormalities (2.88%) (χ^2^ = 166.2, *P* < 0.01).

### Prenatal Ultrasound Screening and Diagnosis of CHD

Before birth, the prenatal ultrasound screening detection rate of CCHD was 97.59%, which was far higher than that of total CHD (51.67%) (χ^2^ = 14.11, *P* < 0.001). Group 1 (simple CHD with no extracardiac abnormalities) had the lowest detection rate before birth, at 35.73%. The ultrasound prenatal diagnosis rate of CCHD was higher than that of simple CHD (χ^2^ = 69.70, *P* < 0.001). The detection rate of CCHD by prenatal screening between 20–24 weeks was 90.98% (968/1,064), which was far higher than that of simple CHD or total CHD (both *P* < 0.001). Moreover, there were more prenatally confirmed cases of CHD plus extracardiac abnormalities than of CHD without extracardiac abnormalities (82.37 vs. 45.80%, χ^2^ = 488.23, *P* < 0.001). The ultrasound prenatal diagnosis rates of CCHD and simple CHD were 91.53% (886/968) and 78.91% (1,291/1,636), respectively (χ^2^ = 69.70, *P* < 0.001). The coincidence rates in the ultrasound main diagnosis of CCHD and simple CHD were 86.79% (769/886) and 92.02% (1,188/1,291), respectively (χ^2^ = 15.23, *P* < 0.001). The details are presented in Table 6.

**Table 6:** Prenatal ultrasound screening and diagnosis of CHD by subgroup.

**Subgroup**	**Total number**	**Diagnosed before birth**	**Diagnosis rate before birth (%)**	**20–24-week ultrasound screening**	**Abnormal results**	**Detection rate (20–24 weeks) (%)**	**Prenatal ultrasound diagnosis**	**Prenatal diagnosis rate**	**Coincident**	**Coincidence rate (%)**
Group 1	4,763	1,702	35.73	4,614	1,168	25.31	864	73.97	770	89.12
Group 2	822	632	76.89	725	468	64.55	427	91.24	418	97.89
Group 3	934	907	97.11	867	782	90.20	716	91.56	610	85.20
Group 4	267	265	99.25	197	186	94.42	170	91.40	159	93.53
Groups 1+2	5,585	2,334	41.79	5,339	1,636	30.64	1,291	78.91	1,188	92.02
Groups 3+4	1,201	1,172	97.59	1,064	968	90.98	886	91.53	769	86.79
Groups 1+3	5,697	2,609	45.80	5,481	1,950	35.58	1,580	81.03	1,380	87.34
Groups 2+4	1,089	897	82.37	922	654	70.93	597	91.28	577	96.65

## Discussion

In this study, we monitored a large sample of pregnant women in the city of Beijing for 3 years to reflect the status of CHD in this population. The incidence of total CHD increased from 10.29‰ in 2018 to 13.45‰ in 2020 and was slightly higher than the global level, i.e., 8–10 ‰ (10); however, the rate of CHD was much lower than that in other studies in China, such as in Zhejiang Province (20.57‰) in 2018 (11). The increasing trend is consistent with most previous reports in China (6) and other countries (10). The increasing incidence of CHD may be due to the following reasons. First, since the fertility policy adjustment, the proportion of older pregnant women has increased, and this change may partially explain the upward trend in the total CHD cases (7), especially in Beijing. Second, the skills of ultrasound screening specialists have continued to improve. Ultrasound screening doctors are trained every year, and provincial administration regularly organizes experts to control the quality of ultrasound screening images and provide guidance. Third, neonatal CHD screening, such as the SpO_2_ test during the neonatal period, has increased. Finally, most importantly, the criteria for the ascertainment of CHD and study populations vary. For example, in the USA, the follow-up time is up to at least 1 year of life or without an age limitation (12). In Southern Israel, the high incidence of CHD may be due to the high rate of consanguinity among local women (13). In our study, the increasing trend in the incidence of CHD may be due to the increased detection of ASD, PDA and VSD. This finding is similar to the results in Guangdong Province in China and other middle-income countries (14). Our study also shows that the proportion of isolated ASD in CHD has an upward trend, which has also been observed in the USA (12) and Europe (15). Our study included ≥3 mm in diameter for ASD and PDA. All cases were followed up within 1 year after birth. Our study also found that the incidence of CCHD did not significantly change throughout the study period. This finding is similar to the international clearing house for Birth Defects Surveillance (~1.9%) (12). Therefore, CCHD is not the main influencing factor of CHD in our research.

In our study, we did not consider the effects of the social or natural environment or other maternal complications. We only analyzed existing data. Maternal age was a risk factor for CHD (16). We found that when the maternal age was more than 35 years, the risk of fetal CHD was high and that the severity of CHD was related to the maternal age. However, data concerning the effect of a younger maternal age on CHD are limited, and the age classification standard also differed. Our age division is consistent with a previously published study investigating a population from Zhejiang Province (11), but the result is inconsistent, the reason may be related to the small sample size of CHD/CCHD in younger pregnant women. Therefore, we should focus on older pregnant women as well as the younger pregnant women. A higher incidence of CHD was found among women without local household registration in our study. While reviewing the literature, we did not find a comparative study based on the classification of household registration but found comparisons based on urban and rural areas. Because the division of urban and rural areas does not apply to Beijing and there were many floating populations, it was more appropriate to classify the participants according to their household registration. It has been reported that urban areas have a higher incidence of CHD, and researchers believe that pregnant women in urban areas have better screening opportunities during pregnancy than those in rural areas (17). However, in Beijing, pregnant women receive medical resources fairly regardless of their household registration. Therefore, we considered that pregnant women with a foreign household registration may not have had knowledge regarding pregnancy preparation before becoming pregnant, did not have pregnancy consultations or did not have oral folic acid and other drugs to prevent birth defects. We also found that women with DM had a higher incidence of CHD, and this result is consistent with previous reports (18). In our study, we also found that if women had CHD babies, the birth gender was a risk factor for CCHD. We found that women who gave birth to a female had a higher incidence of CHD and CCHD. Compared with previous studies, we found that the association between sex and CHD was not consistent, and the reason for this inconsistency is still unclear (19). Some studies have shown that different sex advantages may be related to different classifications of CHD (14).

In our study, the prenatal ultrasound screening detection rate of CCHD was 97.59% before birth, and the detection rate of total CHD was 51.67%. Among the different types of CHD, the prenatal detection rate of simple CHD with no extracardiac abnormalities is the lowest at 35.73%, slightly higher than the 22.8% reported in the previous study (11). This may be related to the different modes of prenatal ultrasound screening. However, the detection rate of CCHD by prenatal ultrasound screening between 20–24 weeks was 90.98%, which is consistent with previous research results, and the detection rate of total CHD was 30.64%. Based on our results, the ultrasound screening mode in Beijing (first trimester, second trimester, and third trimester ultrasound screening) is more effective than single second trimester ultrasound screening. Moreover, we analyzed the main reasons for the low prenatal CHD detection rate and found the following: first, more than 99% of CHD cases diagnosed after birth were simple CHD cases; second, CHD in the neonatal period was diagnosed within 7 days after delivery, which led to the overdiagnosis of CHD. The arterial duct and foramen ovale may not completely develop until 1 year after birth. We also found that the ultrasound prenatal diagnosis rate of CCHD was 91.53%, which is higher than that of simple CHD, which may be related to the fact that families were more concerned about CCHD than CHD. However, the coincidence rate in the ultrasound diagnosis of CCHD was lower than that of simple CHD, which may be an indicator that the ultrasound screening specialist was more able to recognize simple CHD than CCHD; thus, the more common recommendation was that a diagnosing doctor be consulted in cases of possible CCHD.

CHD may be present in combination with extracardiac malformations or genetic diseases or it could be a part of a syndrome (6). Although the accuracy of ultrasound diagnosis has greatly improved, it is still unable to detect all extracardiac malformations. In our study, CHD was isolated by prenatal ultrasound and diagnosed as CHD with other malformations after birth, such as cleft palate and polydactyly. Therefore, CHD fetuses should be closely evaluated to exclude extracardiac abnormalities.

The prevalence of chromosomal abnormalities in CCHD patients was significantly higher than that in CHD patients. The incidence of genetic abnormalities in CHD patients with extracardiac abnormalities was significantly higher than that in CHD patients without extracardiac abnormalities, which is higher than other reports (20). The main reason was that the indications for invasive prenatal diagnosis were different, and Beijing has stricter indications for a prenatal diagnosis. Genetic testing for CHD could provide evidence for pregnancy decision-making. However, due to the lack of congenital cardiologists in most medical institutions, it is difficult to provide authoritative treatment and prognosis consultation in cases of CHD. Therefore, we think that it is necessary to vigorously promote a multidisciplinary cooperation model.

## Conclusion

In Beijing, the high incidence of CHD is due to the high proportion of simple CHD cases. An older or younger age, birth with a non-local household registration and DM during pregnancy should be given more attention. An interventional prenatal genetic diagnosis is recommended for fetuses with CHD plus extracardiac abnormalities to exclude chromosomal abnormalities. The prenatal ultrasound detection rate of CCHD was higher than 97%. Therefore, prenatal ultrasound screening is a highly effective way to detect CCHD, and the first trimester, second trimester, and third trimester ultrasound screening models are better than the single second trimester model. In summary, our findings provide a basis for the formulation of prevention and intervention strategies for CHD.

## Data Availability Statement

The original contributions presented in the study are included in the article/supplementary material, further inquiries can be directed to the corresponding author/s.

## Author Contributions

YZ designed the study, analyzed the data, and wrote the manuscript. WZ collected and sorted the birth defect card information. HX sorted and checked the birth defect data. KL designed the study, guided the article writing, and modified the manuscript. All authors contributed to the article and approved the submitted version.

## Funding

This work was supported by the National Key R&D Program of China (2018YFC1002304).

## Conflict of Interest

The authors declare that the research was conducted in the absence of any commercial or financial relationships that could be construed as a potential conflict of interest.

## Publisher's Note

All claims expressed in this article are solely those of the authors and do not necessarily represent those of their affiliated organizations, or those of the publisher, the editors and the reviewers. Any product that may be evaluated in this article, or claim that may be made by its manufacturer, is not guaranteed or endorsed by the publisher.
